# Editorial: Reaching and Grasping the Multisensory Side of Dexterous Manipulation

**DOI:** 10.3389/fpsyg.2022.866608

**Published:** 2022-03-17

**Authors:** Simone Toma, Luigi F. Cuturi, Marco Santello, Ivan Camponogara

**Affiliations:** ^1^School of Biological and Health Systems Engineering (SBHSE), Arizona State University, Tempe, AZ, United States; ^2^Unit for Visually Impaired People (U-VIP), Italian Institute of Technology (IIT), Genova, Italy; ^3^Dipartimento di Filosofia e Comunicazione, Alma Mater Studiorum, Università di Bologna, Bologna, Italy; ^4^Division of Science, New York University Abu Dhabi, Abu Dhabi, United Arab Emirates

**Keywords:** multisensory integration, reaching, grasping, dexterous manipulation, vision-binocular and stereopsis, haptic (tactile) perception

The wide range of reaching and grasping actions we perform every day stems from the use and integration of multiple sources of sensory inputs within the motor commands. Both sensory and motor signals associated with these goal-directed behaviors provide separate and redundant information necessary to successfully complete object reaching, grasp and manipulation. In this Research Topic we collected a series of original studies investigating such multisensory-motor integration processes. As outlined by the review article by Betti et al., reach-to-grasp movements are affected by the myriad of information we gather through the visual, proprioceptive, auditory, taste, and olfactory senses. Several contributions reported in this review paper revealed how modulating each of the above-mentioned sensory signals affects grasping performance and manipulation performance. Thus, the present Research Topic enriches and expands the existing literature on multisensory-motor integration for reaching, grasping and manipulation by presenting a collection of original research papers addressing five main research areas: (1) the role of haptic and tactile feedback in grasping and manipulation; (2) the effect of visual exploration on grasping and tool use; (3) the relationship between the sensory deficit and multisensory integration; (4) multisensory motor learning; and (5) the neural correlates of visuo-haptic integration in primates.

The role of the haptic feedback in grasping and manipulation has been investigated by Whitwell et al., who showed that advanced knowledge of haptic feedback modulated action performance. Specifically, they used a mirror apparatus where subjects were able to see a “virtual” object reflected in the mirror, spatially coincident with the real one. Subjects had to reach and grasp the object behind the mirror, which could be either physically present or not, with or without concurrent verbal cues signaling the presence (or not) of the physical object. They showed that cueing haptic feedback before performing the action led to a smaller peak of grip aperture than when haptic feedback was uncued and unexpected. This work extended their previous works on visual cueing, showing a flexible use of natural and pantomimed grasping according to the haptic expectation. Concurrently, through the use of a virtual reality setup, Ozana et al. found that withdrawing the haptic feedback at the end of a bimanual grasping task (i.e., not presenting a physical object with matching dimensions of the object presented in virtual reality) increased the hand's aperture variability compared to when haptic feedback was provided. These findings demonstrated that grasping adheres to Weber law only when haptic feedback is absent (pantomimed grasping). Maiello et al. found that haptic feedback is crucial to judge grasp quality. Specifically, grasp quality is better inferred following an active grasp, where subjects had previously experienced the haptic feedback through enclosing the digits on the physical object, than passively viewing the grasping point on the object or watching a video of someone else's grasp on the same object. The role of tactile feedback in object manipulation was investigated by Naceri et al., who found that tactile feedback is fundamental to modulate the forces applied on a lifted object. By reducing the tactile sensitivity through a rubber thimble, they showed an increased grasping force relative to baseline on the covered fingers, demonstrating an independent control of the digits' force when lifting an object.

The effect of visual exploration on grasping and tool use was addressed by Fan et al., who investigated whether surrounding a to-be-grasped object with distractors affected the grasping performance. They found that distractors do not affect object grasping performance, and the latter does not influence object representation. Tamaki et al. investigated two aspects of pre-grasp tool-use behavior. First, these authors asked whether familiarity with an object influences pre-grasp visual exploration, and second, if the intention to use the object as a tool leads participants to shift their gaze on object functional parts (i.e., technical reasoning). Tamaki et al.'s main finding was that technical reasoning increases when the object is only visually explored (without manipulation) and when it is used as a tool, relative to when the object is just lifted. The authors interpreted their findings proposing two different types of technical reasoning associated with tool-use, which they defined as automatic and intentional.

The relationship between the sensory deficit and multisensory integration was thoroughly explored by Bernard-Espina et al., who provided a new theoretical approach to multisensory integration in post-stroke proprioceptive deficits. By merging optimal sensory integration framework with reference frames models (Tagliabue and McIntyre, 2021) they suggested that post-stroke proprioceptive deficits stem from the inability to encode proprioceptive information in a non-joint space. Mencel et al. found that the brain-related activity during a motor imagery training of a patient with bilateral upper limb deficiency in a virtual environment is enhanced compared to healthy individuals and was modulated according to the familiarity with the task. The change of brain activity following motor imagery training was interpreted as training-induced plasticity in patients.

Multisensory-motor learning was addressed by Madan and Singhal, who investigated whether providing tactile and auditory feedback during a visually-guided tapping task improves implicit and explicit learning. They found that while explicit learning benefits from separate unisensory feedback, implicit learning was best when all the sensory modalities were involved. Lastly, by investigating the neural correlates of visuo-haptic integration in non-humans primates Buchwald and Scherberger found that different sensory neural streams subserve grasping performance, even though movement kinematics remained unchanged.

The present Research Topic complements and advances existing literature investigating the variety of contexts where the integration of motor commands with multisensory inputs plays a central role in reaching and grasping. On the one hand, a portion of contributions collected here complements existing literature on behavioral Betti et al. and neurophysiological Buchwald and Scherberger aspects of multisensory integration for object manipulation and motor performance in healthy and motor impaired patients. On the other hand, a second portion of evidence presented here advances the literature of this topic by exploring new research avenues linking grasping behaviors and multisensory integration to grasp quality judgement Maiello et al., grip force modulation Naceri et al., sensory compensation/substitution Bernard-Espina et al., contextual statistics Fan et al., expectations Whitwell et al., motor imagery Mencel et al., tool use Tamaki et al., and motor performance in VR Ozana et al. Due to the wide spectrum of approaches and evidence presented, we believe the present research collection can be of interest for a multidisciplinary audience such as cognitive neuroscientists to engineers.

## Author Contributions

IC and ST wrote the manuscript. IC, ST, LC, and MS revised the manuscript. All authors contributed to the article and approved the submitted version.

## Funding

This material is based upon work supported by the National Science Foundation under Grant No. BCS-1827752.

## Conflict of Interest

The authors declare that the research was conducted in the absence of any commercial or financial relationships that could be construed as a potential conflict of interest.

## Publisher's Note

All claims expressed in this article are solely those of the authors and do not necessarily represent those of their affiliated organizations, or those of the publisher, the editors and the reviewers. Any product that may be evaluated in this article, or claim that may be made by its manufacturer, is not guaranteed or endorsed by the publisher.
